# A151 STRATEGIES TO ENHANCE THE CLINICAL APPLICATIONS OF A NOVEL PH SENSITIVE ANALGESIC IN A PRECLINICAL MODEL OF COLITIS

**DOI:** 10.1093/jcag/gwae059.151

**Published:** 2025-02-10

**Authors:** N Parkar, N Jimenez Vargas, L Blazevic, C Stein, S J Vanner

**Affiliations:** Queen’s University, Kingston, ON, Canada; Queen’s University, Kingston, ON, Canada; Queen’s University, Kingston, ON, Canada; Charité-Universitätsmedizin Berlin, Berlin, Germany; Queen’s University, Kingston, ON, Canada

## Abstract

**Background:**

Improved pain management is an identified top five priority for the treatment of patients suffering from inflammatory bowel disease. Although conventional opioids and NSAIDs can provide pain relief, their side effects pose serious health risks. We developed a unique pH-sensitive analgesic, NFEPP, that is only active at sites of injury/inflammation without affecting healthy tissues, e.g. brain and gut. Preclinical studies using parenteral routes(s.c. or i.v.) demonstrate that NFEPP exhibits potency comparable to fentanyl, but without the risks of addiction or severe side effects like respiratory depression. The duration of action of NFEPP is ~2 hrs, which would require continuous infusion or frequent applications to treat a painful disorder. To broaden the potential clinical applications, strategies are needed to increase its half-life and to understand its efficacy compared to conventional treatments.

**Aims:**

To examine the therapeutic index(TI) of NFEPP and determine if a high index could be exploited to extend its duration of action, and to compare the analgesic efficacy of NFEPP with a standard NSAID, diclofenac.

**Methods:**

Acute colitis was induced in C57BL/6 mice by administering 2.5% dextran sulfate sodium(DSS) in drinking water for five days. Following s.c. injections of vehicle, NFEPP, fentanyl or diclofenac, visceromotor responses (VMRs) to colorectal distension were measured using telemetric transmitters inserted in the abdominal wall muscles. To assess cardiorespiratory side effects, mice were monitored via pulse oximetry.

**Results:**

NFEPP(0.4 mg s.c.) and fentanyl(0.4 mg s.c.) exhibited similar analgesic effects on VMR but fentanyl markedly inhibited O_2_ saturation (~10%; p<0.05), causing hypoxia. NFEPP at 50X’s this analgesic dose(20 mg/kg s.c.) had no effect on O_2_ saturation, suggesting a very high TI. We therefore examined whether the duration of action of NFEPP could be extended into the range of other oral analgesics by increasing the dose (doubling the dose increases T1/2 by ~<1, 1^st^ order kinetics). NFEPP 0.4 mg/kg s.c. was tested at 2 hrs and 1.6 mg/kg s.c. at 6 hrs. NFEPP (1.6 mg/kg s.c.) caused a 37.8% (p<0.05) reduction in VMR responses after 6 hrs, similar to the effect of NFEPP 0.4 mg/kg at 2 hrs (36.8% reduction in VMR; p<0.01). Comparator studies of the efficacy of NFEPP(0.4 mg/kg s.c.; ~EC75 dose) and diclofenac(30 mg/kg s.c., ~EC100 dose) demonstrated a 54.2% (p<0.001) and 49.7% (p<0.01) reduction in VMR, respectively.

**Conclusions:**

NFEPP has a very high TI compared to fentanyl, and this property could potentially be exploited to increase its half-life in oral and/or patch formulations by increasing the dose without risk of respiratory depression. NFEPP has similar or greater analgesic efficacy compared to NSAIDs and could potentially be an alternative for some clinical indications.

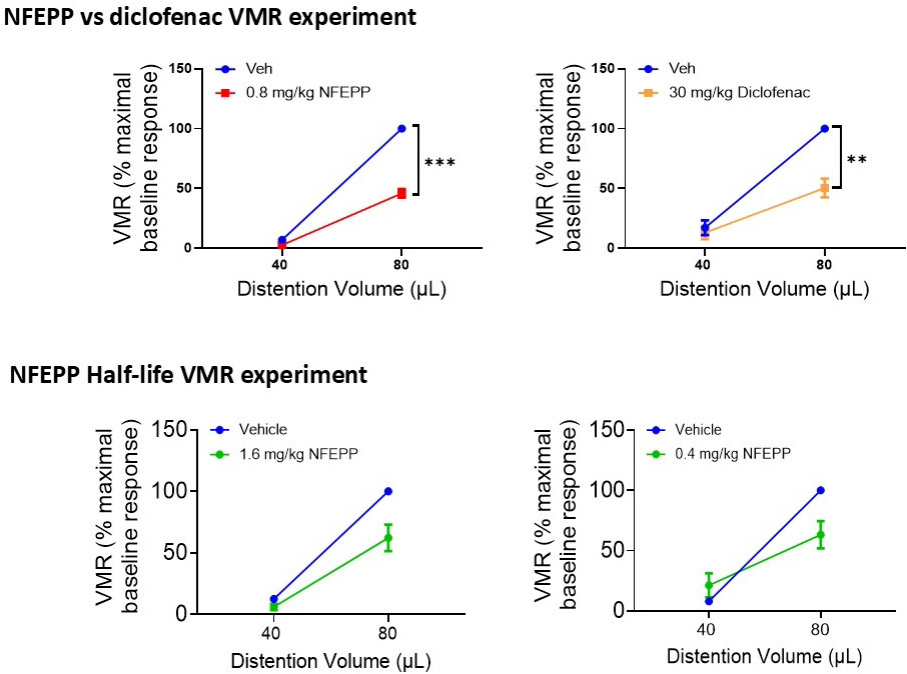

**Funding Agencies:**

CIHR

